# Multifunctionality of *Clausena harmandiana* Extract and Its Active Constituents against Alzheimer’s Disease

**DOI:** 10.3390/cimb44080252

**Published:** 2022-08-15

**Authors:** Chantana Boonyarat, Chavi Yenjai, Orawan Monthakantirat, Rawiwun Kaewamatawong, Pattaporn Poonsawas, Jinda Wangboonskul, Suchada Chaiwiwatrakul, Pornthip Waiwut

**Affiliations:** 1 Faculty of Pharmaceutical Sciences, Khon Kaen University, Khon Kaen 40002, Thailand; 2 Center for Research and Development of Herbal Health Products, Khon Kaen University, Khon Kaen 40002, Thailand; 3 Natural Products Research Unit, Department of Chemistry and Center of Excellence for Innovation in Chemistry, Faculty of Science, Khon Kaen University, Khon Kaen 40002, Thailand; 4 Faculty of Pharmaceutical Sciences, Ubon Ratchathani University, Ubon Ratchathani 34190, Thailand; 5 Faculty of Pharmaceutical Sciences, Thummasart University, Bangkok 10330, Thailand; 6 Faculty of Humanity and Social Sciences, Ubon Ratchathani Rajabhat University, Ubon Ratchathani 34000, Thailand

**Keywords:** dementia, antioxidant, beta-amyloid aggregation, acetylcholinesterase, neuroprotection, scopolamine-induced memory deficit

## Abstract

This study was designed to investigate the effects of the root-bark extract of *Clausena harmandiana* (CH) and its active constituents (nordentatin and 7-methoxyheptaphylline) on pharmacological activities regarding selected targets associated with AD, namely, its antioxidant activity, inhibition of Aβ aggregation, acetylcholinesterase (AChE) activity, and neuroprotective effects. The effect of the CH extract on the cognitive impairment induced by scopolamine was also evaluated in mice. The effects of the CH extract and its active constituents on radical scavenging, Aβ aggregation, and AChE activity were investigated with a 2,2′-azino-bis(3-ethylbenzthiazoline-6-sulfonic acid (ABTS) assay, a thioflavin-T assay, and Ellman’s method. The neuroprotective effects of the extract against hydrogen-peroxide and Aβ toxicity were evaluated with a 3-(4,5-dimethylthiazol-2-yl)-2,5-diphenyl tetrazolium bromide (MTT) assay. In addition, the effects on cognitive impairment induced by scopolamine in mice were evaluated using Morris-water-maze and modified-Y-maze test models. The results of the present study demonstrate that the root-bark extract of CH shows multimodal actions relevant to the AD pathological cascade, including antioxidant effects, the inhibition of Aβ aggregation, the inhibition of AChE function, and neuroprotection against oxidative stress and Aβ toxicity. The extracts could improve both the short- and long-term memory deficits induced by scopolamine in mice.

## 1. Introduction

Alzheimer’s disease (AD) is the most prevalent cause of dementia, characterized by the development of cognitive disfunction and alterations of behavior and social adaptability [1]. AD is a complex progressive disorder with sequentially interacting pathological stages, including the accumulation of β-amyloid (Aβ) with plaque development, the aggregation of the tau protein with the formation of tangles, following with downstream processes such as oxidative stress, cholinergic dysfunction, and inflammation, all of which contribute to synapse loss and neurodegeneration, leading to memory and other cognitive problems [2]. Several therapeutic strategies have emerged over decades, including cholinergic and non-cholinergic drug therapy [3,4]. The current therapeutic options for AD are acetylcholinesterase (AChE) inhibitors including donepezil, rivastigmine, galantamine, and the NMDA-receptor antagonist memantine [5,6]. However, these drugs only represent palliative treatments rather than curing or preventing AD. Thus, searching an effective pharmacotherapy for AD is urgent. Due to the multiplex pathways related to AD pathogenesis, the classical single-target approach that modulates one target may be inadequate. Therefore, searching for candidates that act at multiple points of the pathological cascade is, arguably, a more effective strategy for designing pharmacotherapies to combat AD [7,8,9,10]. Several studies have focused on natural products, which comprise diverse phytochemical constituents showing multiple action or synergistic effects against diseases. Natural compounds from medicinal plants are often safer, with lower levels of adverse effects, than chemically synthesized drugs. Thus, natural products acting on multiple sites of the pathological cascade of AD might provide additional benefits for AD therapy. Therefore, the potential roles of medicinal plants in AD therapy are being widely investigated [11,12,13,14,15,16].

*Clausena harmandiana* (CH) belongs to the Rutaceae family, which is widely distributed in the southeast Asian region. In traditional medicine systems, the young leaves, bark, roots, and flowers of CH are often combined with other herbs to reduce intestinal gas and treat food poisoning [17]. This plant is also used for the treatment of eye pain, headaches, and illness, as well as general health promotion [18,19]. The crude extracts of CH have been reported to exert anti-inflammatory and antioxidant effects in evaluations of their biological activities [20]. Phytochemical investigations have revealed that CH contains a number of carbazole alkaloids and coumarins [21,22,23,24,25]. Several studies have illustrated that coumarins show a wide range of biological activities relevant to AD including antioxidant effects and the inhibition of Aβ aggregation [26], AChE function [27], and iNOS protein expression [28]. Carbazole alkaloids, widely present in various plant species, inhibit platelet aggregation, induce vasorelaxation [29], reduce inflammation, show antioxidant effects, and promote neurogenesis [30]. Based on the activities of its chemical constituents, CH may provide useful therapeutic effects for the treatment of AD.

In this study, we aimed to evaluate the effects of the CH extract and its constituents on the biological activities related to the AD pathological cascade, namely, the antioxidant activity, AChE activity, and inhibition of Aβ aggregation. Additionally, a cell-culture model was used to investigate the neuroprotective effects of the CH extract against oxidative stress and Aβ toxicity. The CH extract was also evaluated for the improvement of memory deficits in mice utilizing modified-Y-maze and Morris-water-maze tests.

## 2. Results

### 2.1. HPLC Fingerprints of the Root Extract of C. harmandiana

**Figure 1:**
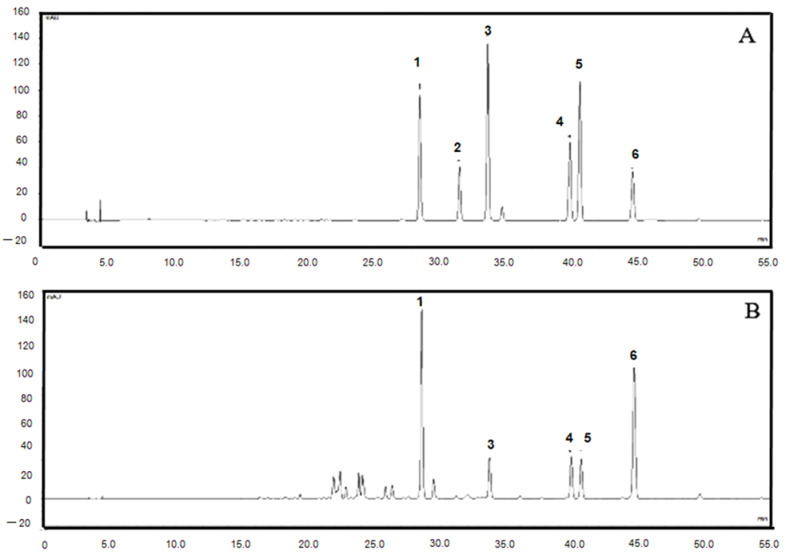
HPLC chromatograms of standards (**A**) and the root extract of *C. harmandiana* (**B**) using a Phenomenex C_18_ column (250 mm × 4.6 mm, 5 μm) and a gradient mobile phase of 0.1% formic acid (**A**) and acetonitrile (**B**) at wavelength 270 nm. Xanthoxyletin (1), osthol (2), nordentatin (3), 7-methoxyheptaphylline (4), heptaphylline (5) and dentatin (6).

The samples were completely separated and eluted in 55 min with total run time 60 min. The chromatograms of crude extract exhibited that the HPLC method effectively separated five standard compounds in these samples without interference from peaks of another compounds. The marker compound peaks in the sample chromatogram were identified by comparison of the retention time with those of standard compounds. The retention time of xanthoxyletin, nordentatin, 7-methoxyheptaphylline, heptaphylline and dentatin were 28.60, 33.73, 39.92, 40.67 and 44.66 min, respectively (Figure 1, Table 1). Peak of osthol was absent in the chromatograms of the root extract of *C. harmandiana*.

### 2.2. In Vitro Antioxidant-Activity Evaluations

Designing AD medicines has been considered with a strong emphasis on the reduction of oxidative stress. We used the ABTS-radical-scavenging method to examine the antioxidant activities of the root-bark extract of CH and its constituents, including nordentatin and 7-methoxyheptaphylline. The ability to scavenge radicals is described by the IC_50_, the concentration of the test compound that resulted in a 50% inhibition of free radicals. Trolox, a water-soluble derivative of vitamin E, was included as a control antioxidant standard. Our results show that the root extract of CH had the ability to remove the ABTS radical with an IC_50_ of 42.21 ± 2.11 µg/mL, as shown in Table 2. Both nordentatin and 7-methoxyheptaphylline demonstrated great ABTS-radical-scavenging capacities. These chemical constituents showed greater radical-removing activity than trolox, the positive control, which had an IC_50_ of 23.67 ± 1.41 µM.

### 2.3. In Vitro AChE-Inhibitory Activity Determination

The effect of the CH extract, nordentatin and 7-methoxyheptaphylline on AChE function was assessed according to the method of Ellman. It is widely known that the anti-dementia effects of tacrine are in consequence of AChE inhibition in the brain [31]. Thus, tacrine was used as a positive control. The results show that the ethanolic extract of CH inhibited AChE activity in a dose-dependent nature, with an IC_50_ of 86.71 ± 5.23 µg/mL (Table 2). The standard reference, tacrine, could inhibit AChE activity with an IC_50_ of 0.36 ± 0.16 µM. Nordentatin, the major constituent of the CH extract, also showed an ability to inhibit the ChE function, with an IC_50_ of 67.79 ± 4.19 µM. The results show that 7-methoxyheptaphylline had no effect against AChE until it reached a concentration of 100 µM. Thus, the inhibitory action on AChE function of the CH extract might partly derive from nordentatin.

### 2.4. Investigation of Aβ-Aggregation Inhibition

The major pathological hallmark of AD is the accumulation of Aβ plaques in the brains [32,33]. The prevention or reduction of Aβ aggregation has been the principal goal of therapeutic strategies under development or in clinical trials [34,35,36]. In this investigation, a Th-T fluorescence assay was used to determine the effects of the CH extract and its constituents on Aβ aggregation. According to Table 2, all the tests inhibited the aggregation of Aβ1-42. The CH extract showed an ability to prevent the aggregation of Aβ1-42 with percent-inhibition values of 65.28 ± 7.54 µg/mL. Nordentatin exhibited better inhibitory activity against Aβ aggregation than 7-methoxyheptaphylline. When compared with curcumin, a reference standard, nordentatin showed greater potency in the reduction of Aβ aggregation.

### 2.5. Effect on H_2_O_2_-Induced Neuronal Cell Death in NG108-15 Cells

**Figure 2:**
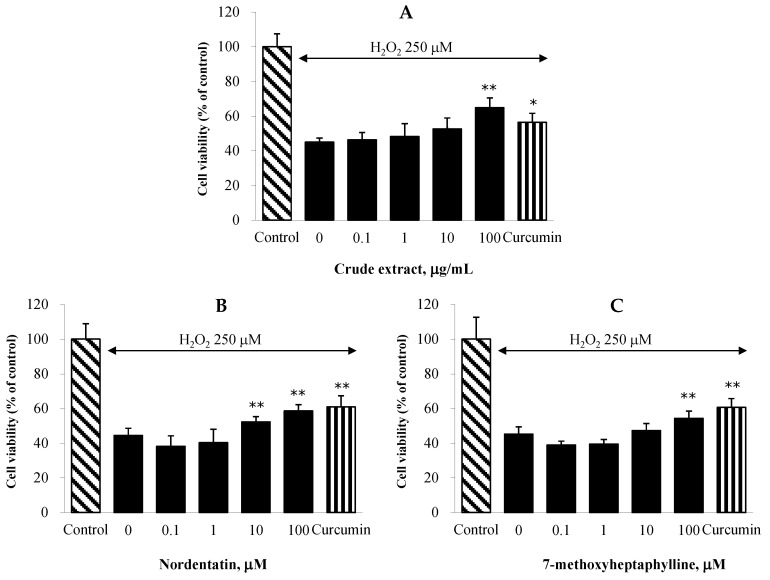
Effect of the CH extract at the concentrations of 0, 0.1, 1, 10, and 100 μg/mL (**A**) and its constituents nordentatin (**B**) and 7-methoxyhepthaphylline (**C**) at the concentrations of 0, 0.1, 1, 10, and 100 μM on H_2_O_2_-induced oxidative cell damage in NG108-15 cells. Curcumin (10 µM) was included as a reference standard. Data are means ± SDs (*n* = 5). Statistical significance was determined by one-way ANOVA. For Turkey’s post hoc analyses: * *p* < 0.05 and ** *p* < 0.01 compared with the H_2_O_2_-treated control group.

The neuroprotection of the CH extract and its active constituents against oxidative stress was evaluated by MTT (3-(4,5-dimethyl-2-thiazolyl)-2,5-diphenyl-2H-tetrazolium bromide) test in a neuroblastoma cell line (NG108-15). Hydrogen peroxide (H_2_O_2_) was applied to provoke oxidative damage, and curcumin was used as a positive control. According to Figure 2, the treatment of the cells with the root extract of CH and its constituents considerably enhanced the cell viability. Nordentatin showed a significant effect at a concentration of 10 μM, and 7-methoxyheptaphylline had a significant impact at 100 μM when compared to the H_2_O_2_-only treated group. The ethanolic extract of CH at a concentration of 100 μg/mL demonstrated the capacity to mitigate the harm caused to neuronal cells by hydrogen peroxide. The neuroprotection against oxidative stress results and the outcome of the in vitro antioxidant test were correlated. This demonstrates that radical-scavenging activity might partly mediate the neuroprotective action of the CH extract.

### 2.6. Effect on Aβ-Induced Neuronal Cell Death in C6 Cells

**Figure 3:**
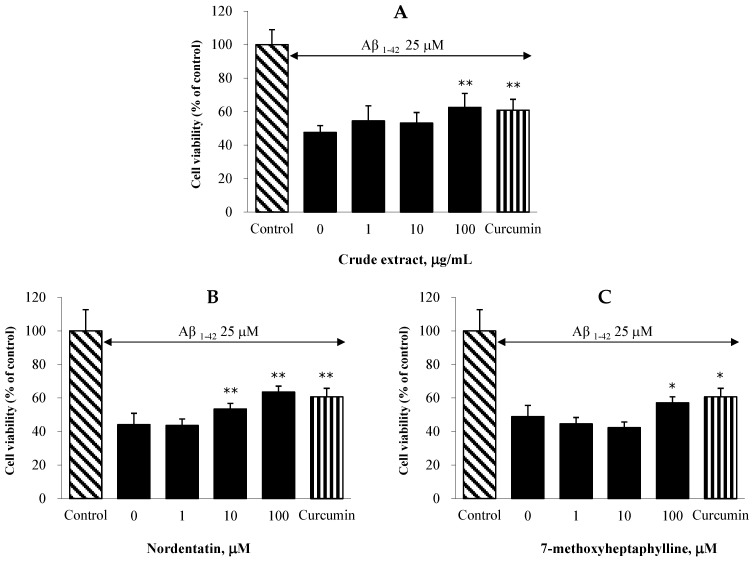
Effects of the CH extract at the concentrations of 0, 1, 10, and 100 μg/mL (**A**), and its constituents nordentatin (**B**) and 7-methoxyhepthaphylline (**C**) at the concentrations of 0, 1, 10, and 100 μM on Aβ1-42-induced neuronal cell death in C6 cells. The values are reported as means ± SDs *(n* = 5). One-way ANOVA followed by the Turkey test, * *p* < 0.05 and ** *p* < 0.01 compared with the Aβ1-42-treated control group.

In C6 astroglioma cells model, the neuroprotective effects of the CH extract and its active ingredients against Aβ1-42 peptide-induced toxicity were assessed. All the test compounds demonstrated neuroprotective properties against neurotoxicity mediated by the Aβ1-42 peptide (Figure 3). The treatment of C6 cells with the CH extract at a concentration of 100 μg/mL considerably decreased the loss of cell viability evoked by the Aβ1-42 peptide. Nordentatin showed a significant effect at 10 μM, and 7-methoxyheptaphylline showed a significant effect at 100 μM compared with the Aβ1-42-peptide-only treated control. The results obtained from an anti-aggregation assay revealed that our extracts could inhibit beta-amyloid aggregation. Thus, the protective action of our extracts against the Aβ1-42 peptide might be attributable to the antioxidant and anti-Aβ-aggregation effects.

### 2.7. The Effect of the CH Extract on the Improvement of a Memory Deficit in Mice Induced by Scopolamine

In the present study, we investigated the effects of the CH extract on scopolamine-induced memory impairments in mice utilizing the Y-maze and Morris-water-maze models. Scopolamine, a nonselective, centrally acting muscarinic-receptor antagonist, blocks cholinergic signaling and produces memory deficits [37]. Thus, scopolamine was used to induce a memory deficit in the mice in this study.

**Figure 4:**
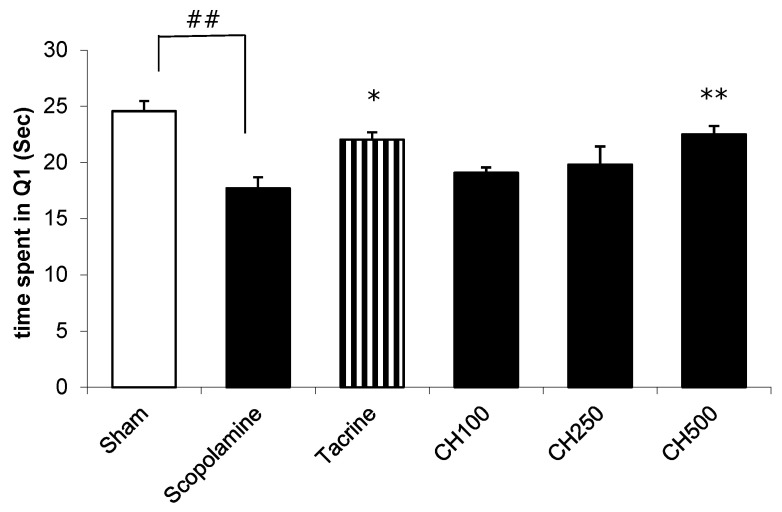
Inhibitory effects of tacrine (at a dose of 10 mg/kg/day) and root-bark extract of CH (at doses of 100 (CH100), 250 (CH250) and 500 (CH500) mg/kg/day on scopolamine-induced-memory impairment according to the water-maze test. The data are represented as means ± SEMs (*n* = 10). One-way ANOVA and Turkey’s post hoc analyses were used for determination of statistical significance. ^##^ *p* < 0.01, a significant difference compared with the controls; * *p* < 0.05 and ** *p* < 0.01, a significant difference compared with the scopolamine-treated mice.

The Morris-water-maze task (MWM) was performed to study the hippocampus-dependent spatial-learning ability. MWM is a model used to assess long-term memory by tracking the escape latency with time [38]. If the animals spent longer swimming time in the target quadrant where the platform had formerly been located during training, this indicated that the mice had greater spatial memory [38]. Figure 4 illustrates the MWM results. The mice in the scopolamine-treated group (means ± SEMs; 17.73 ± 0.94 s) spent significantly shorter time in target quadrant (Q1) than those in the control group (means ± SEMs; 24.56 ± 0.91 s). Pretreatment with tacrine (10 mg/kg), a positive control, improved memory performance; apparently, the longer time was spent in the target quadrant Q1 when compared to the mice in the scopolamine-treated group. Additionally, the animal groups pretreated with the CH extract at a dose of 500 µg/mL (means ± SEMs; 22.52 ± 0.71 s) spent more time swimming in Q1 than the only scopolamine-treated group. The results indicate that the CH extract could improve long-term memory impairment induced by scopolamine in mice.

**Figure 5:**
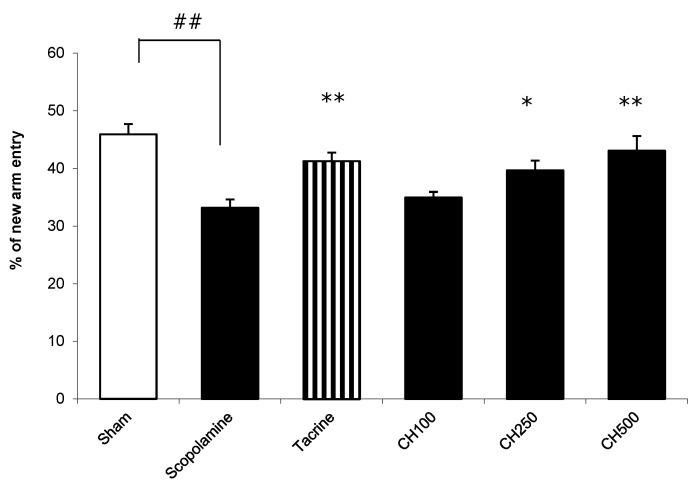
The effect of tacrine (at a dose of 10 mg/kg/day) and root-bark extract of CH (at doses of 100 (CH100), 250 (CH250) and 500 (CH500) mg/kg/day on scopolamine-induced impairment of spatial memory in a modified-Y-maze test. The percentage of time spent by mice in the new arm of a Y-maze task was measured 14 days after chronic extract treatment. The bars indicate means ± SEMs (*n* = 10). One-way ANOVA and Turkey’s post hoc analyses were used for determination of statistical significance. ^##^ *p* < 0.01 versus the control; * and ** *p* < 0.05 and 0.01 versus the scopolamine-treated mice, respectively.

A modified-Y-maze test was carried out to evaluate the short-term spatial working memory. In this task, the total number of arms visited and the percentage of time spent in the new arm were measured. The mice treated with scopolamine exhibited a significant decrease in the percentage of time spent in the new arm compared with the vehicle-treated control mice (scopolamine group, 33.16% ± 1.45%; control group, 45.94% ± 1.77%; Figure 5). When the scopolamine-treated mice were tested 14 days after the initiation of a chronic treatment with the CH extract, the mice treated with the CH extract at doses of 250 and 500 mg/kg/day illustrated a significant increase in the percentage of time spent in the new arm (39.66% ± 1.71%, *p* < 0.05, and 43.06% ± 2.56%, *p* < 0.01, respectively) compared with the scopolamine-treated mice. The results show that the root-bark extract of CH improved the scopolamine-induced impairment of spatial memory in a Y-maze.

Our results suggest that the CH extract has the ability to improve or ameliorate the dysfunction of spatial long-term and working memory in mice induced by scopolamine.

## 3. Discussion

Due to the multi-factorial pathogenesis of AD, agents acting at multiple points of the pathological stage appear to be potential drugs for AD therapy. Natural plants, which have a wide variety of biological and chemical components, may provide multi-target drugs for the treatment of this disease. CH, a traditional medicine in Thailand, was found to be composed of constituents showing several biological activities associated with AD. Thus, CH may have potential multi-target effects against AD.

The root bark of CH has been reported to contain a number of coumarins and carbazoles [21,22,23,24,25]. We standardized the root-bark extract of CH by using three coumarins and four carbazoles [21]. A constituent analysis of the CH extract using HPLC showed that the major active ingredients of the extract were coumarin, nordentatin, and carbazole clausine K [21]. Nordentatin was chosen as a coumarin biomarker in this study. In our activity screening, clausine K showed a weaker inhibitory effect on lipid peroxidation than 7-methoxyheptaphylline. Therefore, 7-methoxyheptaphylline was chosen as a carbazole biomarker in this study.

We investigated the effect of the root-bark extract of CH and its active constituents, including nordentatin and 7-methoxyheptaphylline, on the biological activities related to the AD pathological cascade. The effect of the CH extract on memory-deficit improvement was also evaluated in an animal model.

According to the findings of in vitro experiments, the root-bark extract of CH exhibited a variety of actions relevant to the AD pathological cascade, including antioxidant effects, the inhibition of Aβ aggregation, the inhibition of AChE function and neuroprotection against Aβ toxicity and oxidative stress. The CH root extract and its constituents, 7-methoxyheptaphylline and nordentatin, displayed antioxidant activity and could scavenge the ABTS radicals. Previous studies suggested that a hydroxyl group on aromatic ring influenced the ability to scavenge free radicals [10,39]. The hydroxyl group, an electron donor, promotes antioxidant activity by providing electrons to free radicals. Thus, nordentatin and 7-methoxyheptaphylline, which contain a hydroxy group on the aromatic ring, exhibited the radical scavenging action. However, 7-methoxyheptaphylline possessed weaker activity than nordentatin. The reduction in radical scavenging activity might result from steric hindrance by the bulky substituents neighboring hydroxy group. With regard to AChE inhibitory action, the root extract of CH and nordentatin showed an ability to inhibit AChE function, while 7-methoxyheptaphylline lacked an inhibitory effect. Thus, the inhibitory effect on AChE activity of the CH extract might partly derive from nordentatin. According to recent studies, the coumarin ring is the structure that plays an important role in AChE inhibition by interfering with acetylcholine hydrolysis at the catalytic anionic site [27,40]. This evidence supported the inhibitory activity of nordentatin which has coumarin ring as a core structure. For the effect on the inhibition of Aβ aggregation, the CH extract and its constituents inhibited the aggregation of Aβ1-42. Nordentatin exhibited more potent inhibitory activity against Aβ aggregation than 7-methoxyheptaphylline. Nordentatin showed greater potency in the reduction of Aβ aggregation than curcumin, a reference standard. Furthermore, the root extract of CH and its constituents were able to protect neuronal cell death induced by H_2_O_2_ and Aβ1-42. Our in vitro results illustrated that the ethanolic extract of CH root bark demonstrates multiple modes of action against several targets in the pathological cascade of AD.

It is known that acetylcholine is a crucial neurotransmitter associated with learning and memory. To investigate the impact of the ethanolic extract of CH on memory-deficit improvement, scopolamine was used to induce memory impairment in mice. The effect of the ethanolic extract of CH on the scopolamine-induced memory impairment in mice was investigated by using water-maze and modified-Y-maze tests. It is widely known that scopolamine, a muscarinic antagonist, causes impairment of learning and memory abilities in humans and rodents. This experimental model has been widely utilized to screen for drugs with potential therapeutic benefit in dementia [41,42,43].

In the present study, the systemic administration of scopolamine 30 min before behavioral-test-induced memory impairment was tested using water-maze and modified-Y-maze tests. Scopolamine-induced amnesia was associated with increased oxidative stress and acetylcholinesterase (AChE) activity in mouse brains. The clinically used anti-dementic drug donepezil ameliorated the scopolamine-induced memory impairment by reducing AChE activity and oxidative stress and restoring the cerebral circulation [44]. Chronic pretreatment with the ethanolic root-bark extract of CH reversed the scopolamine-induced memory impairment in mice, as shown by the significant increase in the time spent in Q1 in the water-maze test and the percentage of new arm entries for the modified-Y-maze test. No significant difference among the different groups in locomotor activity was found (data not shown). This excludes the possibility that the locomotor activity contributed to the changes in both behavioral tests. Thus, the in vivo results demonstrated that the CH extract improved both the short- and long-term memory deficits induced by scopolamine in animals.

## 4. Conclusions

The results of the present study indicate that the ethanolic extract of CH root bark shows multimodal activity related to the AD pathological cascade, including antioxidant activity, the inhibition of Aβ aggregation, and acetylcholinesterase inhibition as well as neuroprotection against oxidative stress and beta-amyloid toxicity. The CH extract improved both the short- and long-term memory deficits induced by scopolamine in animals. Thus, CH could serve as a candidate with potential impact for further development in Alzheimer’s treatment. However, the elucidation of the mechanisms of action is an important step in the search for new drug candidates. Thus, further studies focusing on the mechanism of action of CH will be planned in the near future.

## 5. Materials and Methods

### 5.1. Materials

Analytical-grade reagents were purchased from Sigma-Aldrich (SM Chemical supplies Co., Ltd., Bangkok, Thailand), Merck (SM Chemical supplies Co., Ltd., Bangkok, Thailand), and Fluka (SM Chemical supplies Co., Ltd., Bangkok, Thailand) and were used as supplied. All the solvents were routinely distilled prior to use.

### 5.2. Plant Materials and Preparation of the Crude Extract

Root bark of CH was harvested from Khon Kaen province, Thailand. The plant was identified by Mr. Supachai Tiyavorranun, Faculty of Pharmaceutical Sciences, Khon Kaen University. A botanically identified voucher specimen (reference number: 386) was deposited at the Faculty of Pharmaceutical Sciences, Khon Kaen University. The air-dried root bark of CH was ground and macerated with 70% ethanol and periodically stirred at room temperature for 3 d. Concentrated extract was obtained by filtration and then concentration using a rotary evaporator The root bark extract of CH was obtained as a solid brown residue (19.7% yield) and kept in an airtight container at 2–8 °C until use.

### 5.3. HPLC Analysis

The root extract of C. harmandiana was analyzed by a reversed-phase high-performance liquid chromatography system. All analyses were performed on DIONEX 3000 HPLC system (Thermo Fisher, Dreieich, Germany), equipped with LPG 3400 pump and auto sampler (WPS-3000) (EU). Chromatographic separation was performed on a C18 column, 250 mm × 4.6 mm, 5μm (Phenomenex, Torrance, CA, USA). The mixture of 0.1% formic acid (A) and acetonitrile (B) was used as mobile phases in the analysis of xanthoxyletin (1), osthol (2), nordentatin (3), 7 methoxyheptaphylline (4), heptaphylline (5) and dentatin (6) with a flow rate of 0.8 mL/min and monitored at 270 nm. The gradient profile was as follows: 0–5 min, 70% A; 5–30 min, 45% A; 10–20 min, 45% A; 20–30 min, 35% A; 30–40 min, 25% A; 40–50 min, 15% A; 50–60 min, 90% A.

The standard stock solutions were dissolved in methanol at the concentration of 1 mg/mL. Working standard solutions were made by further dilution of stock standard solutions with concentrations of 0.56 to 100 µg/mL. Each 1 mg of crude extract was accurately weighed. The sample was dissolved in methanol and the volume adjusted to 10 mL. The injection volume of sample was 10 µL.

### 5.4. In Vitro Antioxidant Activity Assays [7]

The radical-scavenging activity of the root-bark extract of CH and its constituents, including nordentatin and 7-methoxyheptaphylline, was monitored using the 2,2′-azino-bis(3-ethylbenzthiazoline-6-sulfonic acid (ABTS) method. ABTS was mixed with 2.45 mM potassium persulfate in deionized water and incubated at room temperature for 16 h in the dark. The final product was ABTS radical (ABTS^•+^). The ABTS^•+^ solution was added with ethanol to explore the absorbance of 0.70 ± 0.02 at the wavelength of 734 nm. Afterward, 10 µL of the various concentrations of CH extract or its constituents was mixed with 990 µL of the ABTS^•+^ solution and then kept in the dark for 15 min. Lastly, the absorbance was determined at the wavelength of 734 nm. Trolox was included as the positive control. All determinations were three-times conducted in triplicate wells.

### 5.5. In Vitro Assay of AChE-Inhibitory Activity [45]

The root-bark extract of CH and its constituents were investigated for their AChE-inhibitory activity through Ellman’s spectrophotometric method in a 96-well plate by adding 25 µL of 1 mM acetylthiocholine iodide, which was used as a substrate in the assay, into a mixture of 125 µL of 1 mM 5,5′-dithiobis-(2 nitrobenzoic acid) (DTNB), 25 µL of a 0.1 M phosphate buffer with a pH of 7.4, 25 µL of the CH extract at various concentrations; and 50 µL of 0.2 units/mL AChE from an electric eel (type VI-S). The absorbance at 405 nm was recorded every 30 s for 5 min. The percentage inhibition and the enzyme activity were quantified. The half maximal inhibitory concentration of extract (IC_50_) was appraised graphically from a concentration–inhibition curve for each compound. The test was carried out in independent triplicates.

### 5.6. In Vitro Assay for AB-Aggregation Inhibition Using a Thioflavin-T Assay [46]

A thioflavin-T (ThT) assay was used to detect the aggregation of Aβ1-42. The experiment was carried out as previously described with a minor modification (Quoc Huy et al., 2013). Twenty-five micromolar of Aβ1-42 was prepared in a 50 mM phosphate buffer with a pH of 7.4 and incubated at 37 °C with several concentrations of the CH extract for 48 h. After the incubation, the samples were mixed with a 50 μM glycine/NaOH buffer (pH 9.2) containing 5 μM thioflavin-T. The fluorescence intensities at the 446 nm excitation and 490 nm emission were detected. The percentage inhibition of the aggregation was calculated by following equation: (1-IFi/IFc) * 100%, in which IFi and IFc are the fluorescence intensities of the test sample and control, respectively. All assays were performed at least three times and in triplicate wells.

### 5.7. Effect on H_2_O_2_-Induced Oxidative Cell Damage in NG108-15 Cells [40]

Neuroblastoma cells (NG108-15, ATCC^®^ number HB-12317) were grown in Dulbecco’s modified Eagle medium (DMEM) supplemented with 16 μM thymidine, 100 μM hypoxanthine, 1 μM aminopterin, 1 μg/mL minocycline, and 10% fetal bovine serum. The cultures were maintained at 37 °C in 5% CO_2_. For the assays, the NG108-15 cells were seeded in a 96-well plate and incubated for 48 h. Afterward, the cells were pretreated with various concentrations of the CH extract or its constituents for 2 h. After removing the unabsorbed test compounds, the cells were exposed to H_2_O_2_ for 2 h to induce oxidative stress. The cell viability was assessed by MTT colorimetry. The absorbance at 550 nm was measured in a microplate reader. The experiment was performed in independent triplicates (5 wells/group).

### 5.8. Effect on β-Amyloid-Induced Cell Damage in C6 Cells [40]

Rat C6 cells (ATCC^®^ number CCL-107) were grown in Dulbecco’s modified Eagle medium (DMEM) supplemented with 2 mM L-glutamine, 10% fetal bovine serum, 50 IU/mL penicillin, and 50 g/mL streptomycin. The cell cultures were kept alive at 37 °C in 5% CO_2_. Aβ1-42 (Sigma) at the concentration of 25 μM was incubated at 37 °C for 72 h to form an aggregated amyloid. For the assays, the C6 cells were subcultured into a 96-well plate, in which they were incubated for 24 h. The cells were then exposed to aggregated Aβ1-42 (25 μM) with or without several concentrations of the CH extract or its constituents for 24 h. After that, the cell viability was monitored by staining the cells with 0.5 mg/mL of MTT and measuring the absorbance at 550 nm. All trials were determined in independent triplicates (5 wells/group).

### 5.9. The Effect of the CH Extract on the Memory Deficit in Mice Induced by Scopolamine

Male ICR mice weighing 35–45 g (6 weeks) were obtained from the National Laboratory Animal Centre, Mahidol University, Thailand. All animal experiments were carried out following the guidelines of the Animal Ethics Committee of Khon Kaen University, Thailand (approval number: AEKKU 34/2554). Before testing, the mice were kept in the animal house with 12:12 light–dark cycle for one week in cages of five at a temperature of 25 ± 2 °C and a humidity of 50–55%. The animals had unlimited access to water and food.

The effect of the CH root-bark extract on the memory deficit in mice induced by scopolamine was evaluated utilizing two behavioral models, including modified-Y-maze and water-maze tests. After oral administration for 14 days with an ethanolic extract of CH (100, 250, or 500 mg/kg/day) or a reference standard tacrine (10 mg/kg/day), behavioral changes of ICR mice in learning and memory were investigated. For each experiment, after oral administration of the test compounds for 1 h, memory deficits were induced in the mice using scopolamine. Thirty minutes after the scopolamine injections, all the animals were tested for improvements in the memory deficits using Morris-water-maze and modified-Y-maze tests.

Morris water maze [38]: Determination of the water-maze task was based on the method of Morris. The water maze used a round pool with a size of 28 cm high and 70 cm in diameter. The pool was divided into four quadrants and filled with water (25 ± 1 °C). A platform with a size of 6 cm × 10 cm × 15 cm was located in the middle of a targeted quadrant (Q1) (1 cm under the water surface). The mice were randomly positioned in the pool for the training session and were given 60 s to find the platform. The times finding the platform and escape latency were recorded. To achieve a constant level of escape latency, each animal was given 4 daily trials over 5 days. For the test day, the mice were allowed to swim from each quadrant for 60 s in the pool without the platform. The time spent to swim in target quadrant area (Q1) was recorded.

Modified-Y-maze test [47]: The percentage of time spent in a new arm in a modified-Y-maze task was recorded and evaluated as a short-term spatial-memory task. The apparatus consisted of three identical black polypropylene wall-arms 40 cm in length, with a 12 cm width at the top and a 3 cm width at the bottom, and 18 cm in height. The modified-Y-maze task was performed following the method described by Arunrungvichian. Briefly, the method for a normal-Y-maze test was modified from a one-trial task to a two-trial task with a 30 min intertrial interval. The procedure consisted of 2 phases: a sample phase and a test phase 30 min later. During the sample-phase trial, which was 5 min in duration, one arm was blocked, and the mice were permitted to freely explore between the two open arms. During the 5 min test trial, all the arms were open. The blocked arm that was opened in the test-phase trial was defined as a new arm. The percentage of the time spent in the new arm and the numbers of total arm entries were recorded visually.

### 5.10. Statistical Analysis

Statistical analysis of the data was carried out by a one-way analysis of variance (ANOVA) with Tukey post hoc test. For all the statistical analyses, the significance levels were set at a *p* value < 0.05. Data are expressed as the mean ± SD for the in vitro and mean ± SEM for the vivo experiments.

## Figures and Tables

**Table 1:** The retention time of five marker compounds in CH root extract.

Chemical Constituents in CH Root Extract	Structure	Retention Time (min)
xanthoxyletin	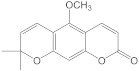	28.60
nordentatin	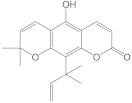	33.73
7-methoxyheptaphylline	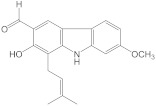	39.92
heptaphylline	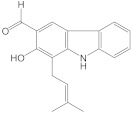	40.67
dentatin	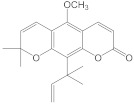	44.66

**Table 2:** The effects of the CH root extract and its constituents on ABTS-radical scavenging, AChE activity, and Aβ aggregation. All determinations were assessed in triplicate wells at least three times.

Compounds	ABTS Assay ^a^ (IC_50_)	AChE Assay ^b^ (IC_50_)	Aβ-Aggregation Assay ^c^ (% Inhibition)
Tacrine	nd	0.36 ± 0.16	nd
Trolox (µM)	23.67 ± 1.41	nd	nd
Curcumin (10 µM)	nd	nd	44.86 ± 6.90
CH extract (µg/mL)	42.21 ± 2.11	86.71 ± 5.23	65.28 ± 7.54
7-methoxyheptaphyline (µM)	10.78 ± 0.11	>100 µM	18.01 ± 5.72
Nordentatin (µM)	3.78 ± 0.13	67.79 ± 4.19	75.34 ± 4.47

nd, not determined. ^a^ Data are represented as IC_50_, the concentration of the test substance that inhibited free radicals by 50% (mean ± SD). ^b^ Data are represented as IC_50_, the concentration of the test substance that inhibited AChE activity by 50% (mean ± SD). ^c^ Data are represented as the percent inhibition at the concentration of 100 µg/mL for the CH extract and 10 µM for its constituents.

## Data Availability

The data presented in this study are available on request from the corresponding authors.
